# Postoperative Endophthalmitis After Cataract Surgery: An Update

**DOI:** 10.7759/cureus.22003

**Published:** 2022-02-08

**Authors:** Saad Althiabi, Abdulaziz J Aljbreen, Asma Alshutily, Faisal A Althwiny

**Affiliations:** 1 Ophthalmology, Unaizah College of Medicine, Qassim University, Unaizah, SAU

**Keywords:** prophylaxis, cataract surgery, postoperative, endoophthalmitis, cataract

## Abstract

Postoperative endophthalmitis is a serious complication that can happen after cataract surgery. It occurs mainly due to invasion of the globe by microbial flora, bacteria, or fungi from the adnexa and environment during the time of surgery. All patients undergoing cataract surgery should be evaluated for any potential risk factors that can enhance the development of postoperative endophthalmitis; managing the intraoperative risk and prophylaxis protocols should be considered in order to reduce the risk of endophthalmitis. Early follow-up after cataract surgery is highly recommended to detect any sign of endophthalmitis so as to treat it immediately and ensure patient compliance on post-surgery medication and precautions to reduce the serious complications caused by late diagnosis and treatment of post-cataract endophthalmitis.

## Introduction and background

Although the high safety of cataract surgery and the promising visual outcome, postsurgical complications do arise even with the best hand. As cataract surgery represents a large segment of intraocular operations, most scientific reports concerning postoperative endophthalmitis focus mainly on cataract surgery [1].

One of the most feared complications after cataract surgery is the increased vulnerability to postoperative endophthalmitis. This mostly starts during cataract surgery where a corneal incision is made in the anterior chamber of the eye to remove the cataractous lens, which permits the entry of ocular surface fluid containing bacterial flora. Of postoperative endophthalmitis cases, 15-30% have a grave prognosis on the visual outcome and medical care expenditures [2]. The unfavorable visual outcomes of postoperative endophthalmitis in cataract surgery ranges from decreased visual acuity less than 20/200 to the loss of the eye. There is no doubt that endophthalmitis elicits distress and loss of quality of patient life in addition to the heavy load on the healthcare organizations as it often necessitates further hospitalization and even surgical intervention [1].

## Review

Epidemiology

The exact incidence of postoperative endophthalmitis in cataract surgery is debatable as it is an infrequent complication of cataract surgery. In the United States, scientific reports described an incidence rate of 0.247% from 1998 to 2001 [3]. Taban et al. reported an overall endophthalmitis rate of 0.265% worldwide after a retrospective meta-analysis of the published literature between 2000-2003 [4]. In Europe, the European Society of Cataract & Refractive Surgeons (ESCRS) described a rate ranging from 0.049% to as high as 0.345% in the control group after a large, randomized clinical trial of antimicrobial prophylaxis from 2003 to 2006 [5]. 

Risk factors

The severity and outcome of postoperative endophthalmitis in cataract surgery mainly depend on several risk factors related to patients’ demographics such as old age, especially males with doubled risk ratio, rural residence, and immunosuppressive conditions such as diabetes mellitus [6]. Blepharitis, ectropion, and conditions involving an increased number of ocular bacteria are associated with an increased risk of developing postoperative endophthalmitis.

Intraoperative risk factors were reported with intracapsular cataract surgery, vitreous loss, and anterior vitrectomy. Also, the rates of endophthalmitis vary according to the intraocular lens (IOL) optic material as postoperative endophthalmitis was reported to have increased incidence with silicone and polymethyl methacrylate (PMMA) compared to acrylic one with four folds of increased ratios [4]. On the other hand, insertion of IOL using a sterile injector reported a lower rate of endophthalmitis than in IOL insertion with forceps. This theory may be confounded by associated wound construction [4]. Moreover, the virulence factors, the infectious dose of inoculated pathogens, and their antibiotic sensitivity play a major role in the prognosis of endophthalmitis [7]. 

The wound structure determines the potential passage of microbes from the ocular surface into the anterior chamber. Laboratory studies reported that unsutured clear corneal incisions are water permeable and allow the influx of extraocular fluids, which may raise the rate of endophthalmitis postoperatively [4]. Furthermore, a double fold to triple fold increased risk of postoperative endophthalmitis is accompanied by surgical complications, specifically posterior capsular rupture [8]. This association is probably related to the increased use of surgical instrumentation and prolonged operative time, which further increased exposure to the extraocular bacterial flora. Based on recent investigations, late application of topical antibiotics a day after surgery instead of the same day of operation and usage of older generations of fluoroquinolone antibiotics raise the risk of postoperative endophthalmitis [9].

Clinical presentation

Postoperative endophthalmitis occurs mainly due to invasion of the globe by microbial flora, bacteria, or fungi from the adnexa and environment during the time of cataract surgery. Endophthalmitis is clinically presented by decreased visual acuity with pain and redness of the eye, which usually occurs within one to two weeks postoperatively. 

Chronic postoperative endophthalmitis may, in rare cases, occur months after the surgery [6]. Despite the great advances in the management of postoperative endophthalmitis, the prognosis is not usually favorable due to associated poor visual outcomes as less than 50% of the endophthalmitis patients are able to achieve a final visual acuity of 20/40 or better [1]. On the other hand, the prognosis is usually compromised by the increased incidence of bacterial resistance even with the growing era of optimal disinfectants and antibiotic delivery strategies [1]. 

Pathogenic organisms

There is no doubt that a clear understanding of the causative organisms is an essential step in the prophylaxis against endophthalmitis. According to a study by Pijl et al., gram-positive coagulase-negative *Staphylococcus* (70% of the cases) is the most incriminated causal bacteria of postoperative endophthalmitis giving symptoms within the first weak postoperatively [10]. Gram-positive coagulase-negative *Staphylococcus* expressed more than 90% sensitivity to vancomycin, cefotaxime, levofloxacin, imipenem, meropenem, and most aminoglycosides except neomycin. However, the emergence of methicillin-resistant *Staphylococcus aureus* (MRSA) and methicillin-resistant *Staphylococcus epidermidis* (MRSE), which are resistant to even the fourth-generation fluoroquinolones, makes the management of this complication a very difficult mission [11]. Also, *Klebsiella pneumonia* was reported to induce postoperative endophthalmitis after cataract surgery in Southeast Asia, especially in elderly diabetic patients [6]. Unfortunately, *Enterococci* has been verified as a causal pathogen due to its relative resistance to cefuroxime; dormant low-virulent *Propionibacterium acnes* in the posterior capsule has also been claimed to develop a milder, chronic course that may be unnoticed, and it was reported to be responsible for 41-63% of cases of chronic postoperative endophthalmitis after cataract surgery [12]. *Candida albicans* was also recorded to be one of the most commonly isolated fungi giving a variable presentation and accounting for 16-27% of cases [12].

Prophylaxis protocols

Numerous perioperative procedures have been attempted to avoid this serious complication [1]. Lately, prophylaxis against postoperative endophthalmitis has progressed extensively. Meticulous surgical preparation including lid hygiene to reduce conjunctival flora is greatly recommended in the periorbital area and conjunctival sac. Preoperative copious irrigation by topical antiseptic povidone-iodine and chlorhexidine in the periocular area are well-thought-out to be the cornerstone in postoperative endophthalmitis preclusion. However, the possible corneal toxicity by chlorhexidine restricts its application in most settings [13]. Most cases of post-cataract endophthalmitis are caused mainly by the periocular bacterial flora since microorganisms in the tear film can invade the anterior chamber through surgical incisions during the operation as well as the early postoperative period [14]. Furthermore, the diverse antibiotic prophylaxis protocols are another commonly proposed preventive measure with different administration routes (topical, intraocular, subconjunctival, oral, intravenous), and timing (preoperative, intraoperative, perioperative, postoperative) [15]. Unlike antiseptics, antibiotics specifically target biological pathways such as replicative, synthetic, or metabolic enzymes of the microbial pathogens, with a safer introduction to living tissues but they require time to convey their effects.

Aminoglycosides, cephalosporins, fluoroquinolones, and chloramphenicol are the most suggested antibiotics in such protocols [16]. Moreover, the systematic use of intracameral cefuroxime was recommended by a prospective randomized controlled trial of postoperative endophthalmitis prophylaxis conducted by the European Society of Cataract & Refractive Surgeons (ESCRS). Intraoperative intracameral antibiotic administration is a highly efficient way to achieve high intraocular antibacterial coverage with optimal antibiotic concentrations for pathogens introduced into the eye during the cataract operation [7]. Experimental studies proved that subconjunctival injection is highly efficient in antibiotic delivery for preventing post-cataract endophthalmitis perioperatively. Subconjunctival injections of vancomycin and ceftazidime can achieve adequate aqueous concentrations to reduce the risk of post-cataract endophthalmitis, despite the insufficient vitreous penetration. However, it is sometimes used nowadays [17]. Moreover, it was proved that a three-day course of antibiotics is more effective than a one-day or one-hour course, especially with the synergistic effects of povidone-iodine [18]. This recommendation coincides with those of retrospective studies from Spain that confirmed the significant effect of systematic intracameral cefuroxime injections in post-cataract endophthalmitis prophylaxis with growing and compelling evidence of its success relative to its price [15]. High to moderate evidence of post-cataract endophthalmitis risk reduction by intracameral application of cefuroxime, cefazolin, and moxifloxacin was verified in a systematic review meta-analysis conducted by Kessel et al. [19]. 

Povidone-iodine

Povidone-iodine displays a wide range of microbicidal activity against bacteria, fungi, protozoa, and viruses. They have a nonselective topical killing effect on microbes by disrupting cellular membranes. Numerous prospective studies have verified povidone-iodine as the only topical prophylactic antiseptic known to reduce endophthalmitis perioperatively with a three-fold to five-fold reduction rate due to its powerful reduction of conjunctival bacterial concentration with 96.7% bactericidal activity within one minute of irrigation [20]. Although the most applied concentration of povidone-iodine is 5%, there is no universal consensus on its concentration, period, and timing of application. Povidone-iodine is widely used in almost all ocular operations except in patients with hypersensitivity reactions [21].

Cefuroxime

Cefuroxime is the most widely used antibiotic during cataract surgery [22]. It is a second-generation cephalosporin that inhibits the formation of the peptidoglycan layer of the bacterial cell wall. Lower post-cataract surgery endophthalmitis rates with intracameral cefuroxime injection were reported in several retrospective and epidemiologic studies [22]. Unfortunately, cefuroxime is ineffective against MRSA and *Enterococci* [6].

Cefuroxime is registered in 24 European countries; it has not been approved by the FDA for intracameral prevention of post-cataract surgery endophthalmitis. It is formulated into single-use syringes (1 mg in 0.1 ml) for intraoperative use [22]. 

Intracameral cefuroxime has a wide safety margin and it is well-tolerated as reported by most studies with few associated side effects. Hypersensitivity reactions are fortunately uncommon; however, anaphylactic reactions (urticaria, bronchoconstriction, or life-threatening circulatory reactions) were recorded after intracameral cefuroxime application in patients with penicillin allergies [23]. The most described side effects resulting from accidental intracameral cefuroxime overdoses comprise macular edema, hemorrhagic retinopathy, serous retinal detachment, and corneal edema [24].

Moxifloxacin

Notably, moxifloxacin (a fourth-generation fluoroquinolone) possesses a very powerful concentration-dependent bactericidal effect on both gram-positive and gram-negative bacteria, as well as a considerable killing effect on atypical organisms. Moxifloxacin expresses broad-spectrum microbial coverage with a very good diffusion into the anterior chamber, and low adverse effects [25]. However, coagulase-negative staphylococci expressed increasing fluoroquinolone resistance, which can be overcome by increasing the administered intracameral dose [26]. Moxifloxacin inhibits topoisomerase I (DNA gyrase) and topoisomerase IV, enzymes required for replication, transcription, and repair of bacterial DNA during bacterial cell division [25]. Although the application of intracameral moxifloxacin has not been investigated in randomized controlled trials, observational studies confirmed its efficacy with the overall reduction of endophthalmitis after cataract removal [27].

Moxifloxacin as a preservative-free formulation can be used either diluted or not for intracameral injection according to the concentration needed [28]. It has a very wide safety margin with no reported toxic effects on corneal endothelial cells, trabecular cells, and retinal pigment epithelial cells, even with previous exposure to inflammation and oxidative stress. However, allergic and anaphylactic reactions have been reported with preoperative topical application of moxifloxacin but hypersensitivity reactions are still very rare [29].

Vancomycin

The glycopeptide vancomycin antibacterial mechanism of action depends on binding to pentapeptides to prevent peptidoglycan polymerization, and hence weakens the bacterial cell wall. Although vancomycin has powerful bactericidal activity against *S. epidermidis*, *S. aureus* (both methicillin-sensitive and methicillin-resistant strains), and most strains of *Streptococcus*, it expresses a very weak effect on gram-negative bacteria, including the pseudomonads. Recently, intracameral vancomycin has been greatly recommended by the American Society of Cataract and Refractive Surgery for endophthalmitis prophylaxis during cataract surgery [30].

Up till now, there is no prepared, single-use syringe for intraocular application of vancomycin. Therefore, in order to apply it intracameral, vancomycin powder 500 mg and 1000 mg vials are reconstituted by dissolving the powder in a balanced salt solution to give a final concentration of 10 mg/1 ml from which 0.1 ml (1 mg) can be injected intracamerally [31]. Application of vancomycin intracamerally has a wide safety margin. Nevertheless, cases of severe, bilateral, ischemic retinal vasculitis and hemorrhagic occlusive retinal vasculitis have been reported in the form of delayed-onset, painless vision loss following cataract surgery. Vasculitis is supposed to be a type III hypersensitivity reaction rather than direct drug toxicity [32]. 

Despite the powerful effect of vancomycin on *S. epidermidis* and *S. aureus*, its routine use in postoperative prophylaxis of endophthalmitis should be restricted except in patients with life-threatening allergies to beta-lactam antibiotics. Vancomycin can be administrated either intravenously, as an intracameral injection, or topically as an irrigating solution [33]. Vancomycin is proposed to be the antibiotic of choice for both prophylactic and therapeutic intracameral injections of endophthalmitis in many parts of the world with 100% sensitivity of the gram-positive bacteria [34].

Corticosteroid Therapy

Topical corticosteroid therapy is believed to offer help in postoperative endophthalmitis management in order to reduce the immune-inflammatory response with the subsequent tissue damage. The antimicrobial alone may induce aggravating inflammatory responses to dead organisms plus those induced by the living organisms. Therefore, corticosteroids are strongly indicated in collaboration with optimal antibiotic cover to reduce the anterior segment inflammation except in cases of suspected fungal infection. Corticosteroids suppress inflammation by inhibition of macrophages and neutrophil migration, as well as inhibition of phospholipase A2 leading to decreased prostaglandin synthesis and reduced capillary permeability. Furthermore, stabilization of lysosomal membranes by corticosteroids inhibits neutrophils, mast cells, macrophages, and basophils degranulation [19].

The most practicable choice is the instillation of topical prednisolone acetate 1% for several days up to several weeks. Subconjunctival injection of 4 mg dexamethasone is administered at the time of initial intravitreal antibiotic therapy. Oral corticosteroid (prednisone 30 mg twice daily for 5-10 days), is also recommended beginning postoperative day one. However, the timing, dosing, and route of steroid administration are controversial and highly dependent on the organism responsible. [33].

Diagnosis

**Figure 1:**
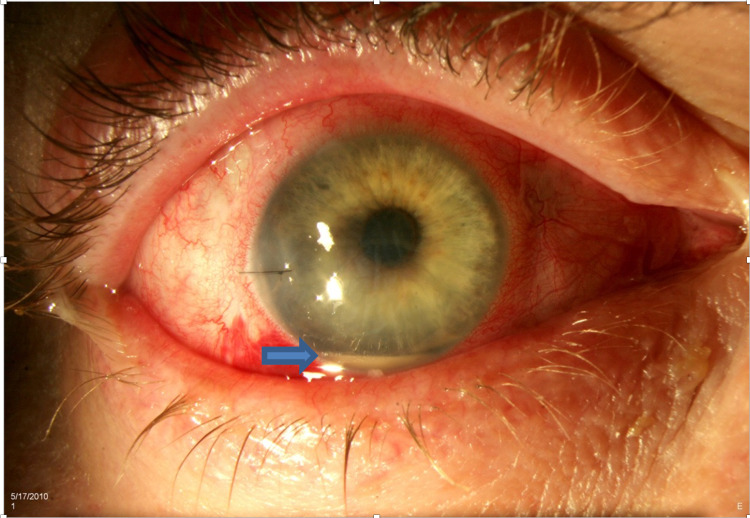
Acute-onset postoperative endophthalmitis with sutured corneal wound and hypopyon (arrow) Source: Vaziri et al. [36]

Diagnosis of post-cataract endophthalmitis depends chiefly on clinical suspicion. Signs and symptoms include hypopyon (Figure 1), hazy media, light perception (LP) vision only, blurred vision, red eye, pain, swollen lid, and photophobia, which can then be confirmed by the sampling of aqueous and vitreous as soon as possible [2]. It is important to consider the potential differential diagnosis of endophthalmitis, including noninfectious inflammation (toxic anterior segment syndrome), residual lens material, flare-ups of previous uveitis, and vitreous hemorrhage. Toxic anterior segment syndrome (TASS) mimics infective endophthalmitis due to the various toxins entering the eye during the operation leading to severe corneal edema. All these conditions are associated with obvious inflammatory reactions and hypopyon may clinically mimic infective endophthalmitis. In order to differentiate between TASS and infective endophthalmitis, It should be kept in mind that TASS is associated with a hypopyon and fibrin formation in the anterior chamber without the involvement of the vitreous as is in the case with infective endophthalmitis [35]. Isolating the causative organism for gram staining, culture, and polymerase chain reaction (PCR) test is the mainstay in guiding treatment in resistant cases to assess antibiotic susceptibility, even though the treatment of suspected cases with empirical broad-spectrum antibiotics should be started at once [36]. B-scan ultrasonography is recommended especially in culture-negative cases, which occur in approximately 30% of cases. It also can measure the degree of vitreous opacification and decide the presence of both choroidal and retinal detachment [37]. 

Management

Acute Postoperative Endophthalmitis 

Systemic antibiotics have a controversial role in the treatment of postoperative endophthalmitis. The systemic amikacin and ceftazidime were reported to have no effect on the final visual outcome this is because these drugs fail to cross the blood ocular barrier [36]. On the other hand, systemic ciprofloxacin versus moxifloxacin had reported a faster resolution of hypopyon and a decreased need for repeat intravitreal antibiotics in patients with acute postoperative endophthalmitis because these drugs cross the blood ocular barrier [38]. 

The cornerstone of the control of massive bacterial postoperative endophthalmitis is the simultaneous intravitreal injection of unpreserved dexamethasone and broad-spectrum antibiotics combination [19]. Vancomycin-ceftazidime is injected intravitreally as first-line treatment, and vancomycin-amikacin is used as a second-line [39]. Each agent is diluted in 0.1 mL of sterile water or saline. Ceftazidime is preferred over amikacin because of the small risk of macular infarction with injected aminoglycosides. Antibiotic concentrations in the vitreous decline rapidly following injection and most last only 24 to 48 hours. Thus, one injection of antibiotics may not maintain levels in the vitreous long enough to kill all bacteria. Repeat injection of vancomycin or ceftazidime may be indicated after 48 hours if there is persistent or worsening intraocular inflammation; the second injection of amikacin is avoided given concerns for retinal toxicity. The choice of antibiotic for repeat intravitreal injection is based on the culture result. [39]. However, the ESCRS emphasizes administrating adjunctive systemic antibiotic therapy for the management of acute postoperative endophthalmitis [36]. In cases of fulminant refractory acute postoperative surgery endophthalmitis with rapidly worsening visual acuity, complete vitrectomy operation is the fundamental option to debride ocular pus [7].

Delayed-Onset Postoperative Endophthalmitis

**Figure 2:**
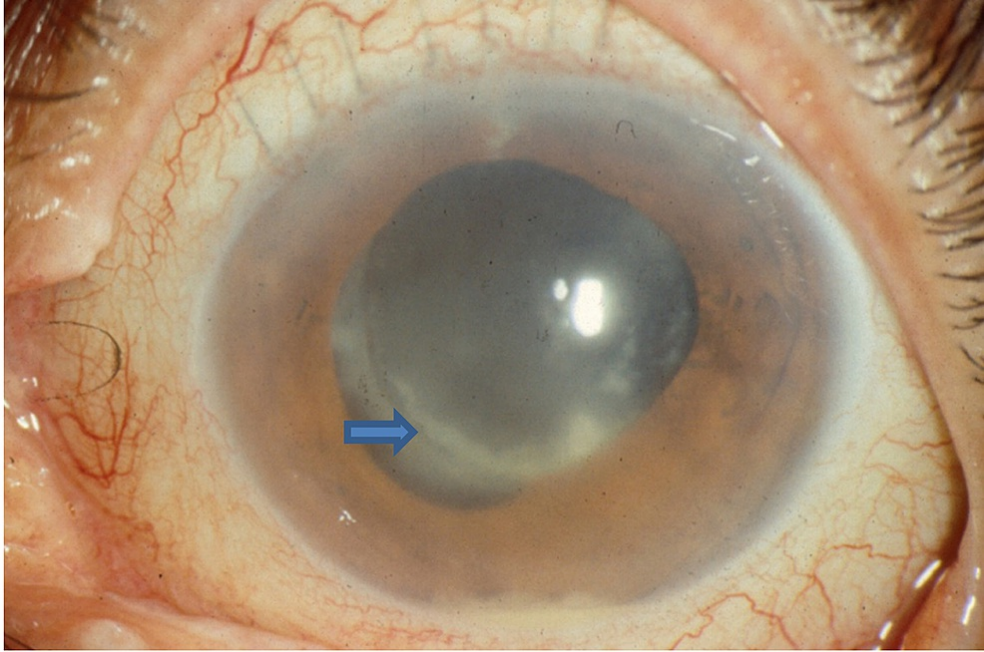
Delayed-onset (chronic) postoperative endophthalmitis with small hypopyon and peripheral intracapsular infiltrates (arrow). Source: Vaziri et al. [36]

The treatment of chronic postoperative endophthalmitis is difficult because of delayed diagnosis due to variable clinical manifestations (Figure 2) with different differential diagnoses as well as different causative organisms [40]. Treatment usually involves partial capsulectomy associated with injection of intraocular antibiotics, but this regimen may lead to complete resolution in only 50% of the cases. Additionally, 70-90% of the cases were completely treated with total capsulectomy and IOL exchange or removal because the organisms were sequestered in the capsule [40]. Therefore, intravitreal antibiotics injection, IOL extraction, and vitrectomy remain the best treatment options [41]. 

## Conclusions

In order to reduce the risk of postoperative endophthalmitis, all patients undergoing cataract surgery should be evaluated for any potential risk factors. At the time of surgery, 5% povidone-iodine is the antiseptic agent of choice for the area surrounding the eye as well as the ocular surface. Meticulous application of intracameral antibiotics in a sterile manner at the end of the cataract operation is a necessary approach with regard to the antibiotic type, safety profile, and potential complications. Moreover, early follow-up after cataract surgery is highly recommended to detect any sign of endophthalmitis and treat it immediately and ensure patient compliance on post-surgery medication and precautions to reduce the serious complications caused by late diagnosis and treatment of postoperative endophthalmitis. 
